# Spontaneous Type I IFN response in SAMHD1-deficient mice requires both, functional intracellular RNA and DNA sensing pathways

**DOI:** 10.1186/1546-0096-13-S1-O33

**Published:** 2015-09-28

**Authors:** R Behrendt, T Schumann, A Gerbaulet, S Wittmann, T Gramberg, A Roers

**Affiliations:** 1 Medical Faculty Carl Gustav Carus TU-Dresden, Institute for Immunology, Dresden, Germany; 2 Friedrich-Alexander University Erlangen-Nürnberg, Institute of Clinical and Molecular Virology, Erlangen, Germany

## Introduction

Nucleic acids are potent inducers of the antiviral type I interferons (IFN) and therefore represent prototypic PAMPs in viral infections. Innate nucleic acids sensors patrol the cytoplasm for the presence of RNA and DNA but have only a limited capacity to discriminate between endogenous and exogenous nucleic acids. Therefore mechanisms evolved that prevent the accumulation of endogenously derived nucleic acids. In Aicardi-Goutières syndrome (AGS), which represents a rare monogenic variant of the prototypic autoimmune disease systemic lupus erythematosus, genetic defects of these mechanisms result in production of large amounts of type I IFN. SAMHD1 is an intracellular nuclease that degrades RNA and DNA and cleaves deoxynucleotides (dNTP) into nucleosides and inorganic triphosphate, mutation of which cause AGS. The nucleotide triphosphohydrolase also confers antiretroviral activity to SAMHD1, as dNTP degradation was shown to represent a major block to reverse transcription of retroviruses. How and why SAMHD1-deficent cells activate the type I IFN system is not known so far. Furthermore, a comprehensive analysis of the antiretroviral potential of mouse SAMHD1 is still lacking.

## Objectives

We aim to identify essential pathways that mediate the spontaneous type I IFN response in SAMHD1 knockout mice.

## Materials and methods

We generated SAMHD1 knock out mice and in parallel inactivated crucial molecules of the type I IFN systems in these mice. Activation of the type I IFN system was quantified by global transcriptome sequencing. Antiretroviral activity of murine SAMHD1 was assessed by infection of mutant mice with GFP-Reporter retroviruses.

## Results

SAMHD1-deficient mice do not develop any signs of inflammation or systemic autoimmunity. However, they spontaneously produce IFNb that subsequently activates transcription of interferon-stimulated genes (ISG). This response was abolished in SAMHD1 IFNb and in SAMHD1 IFNAR1 double deficient mice. Surprisingly, additional inactivation of both, the intracellular RNA and DNA sensing machinery, by knocking out MAVS or STING in SAMHD1-deficient mice, respectively, suppressed the spontaneous IFN production. In vivo infection experiments showed that single deficiencies of IFNAR1 and SAMHD1 only slightly or moderately increased reverse transcription, respectively. Interestingly, in SAMHD1 IFNAR1 double deficient mice we found reverse transcription increased by an order of magnitude compared to wild type and the single deficient mice.

## Conclusion

In contrast to the situation in other AGS-mouse models, in SAMHD1-deficient mice both, a functional intracellular RNA and DNA sensing machinery are required to spontaneously activate the type IFN system. Understanding the mechanisms that establish the chronic IFN response in this model will be instrumental to elucidate whether there is a unifying concept underlying the pathogenesis of AGS.

